# MicroRNAs in Neurodevelopment and Their Relationship with Neurological, Neuropsychiatric and Neurodegenerative Diseases

**DOI:** 10.3390/ijms27219539

**Published:** 2026-10-25

**Authors:** Leonardo Catalano-Iniesta, David Díez-Castro, Josefa García-Barrado, Marta Carretero-Hernández, David Hernández-González, Teresa Vicente-García, Luis Torres, José Carretero

**Affiliations:** 1 Department of Human Anatomy and Histology, Faculty of Medicine, University of Salamanca, 37007 Salamanca, Spain; leonardo.catalano@usal.es (L.C.-I.); dadicas@usal.es (D.D.-C.); mcar@usal.es (M.C.-H.); davidhnz@usal.es (D.H.-G.); 2 Laboratory of Neuroendocrinology, Institute of Neuroscience of Castilla y León, University of Salamanca, 37007 Salamanca, Spain; barrado@usal.es; 3 Laboratory of Neuroendocrinology and Obesity, Biosanitary Institute of Salamanca (IBSAL), 37007 Salamanca, Spain; 4 Department of Physiology and Pharmacology, Faculty of Medicine, University of Salamanca, 37007 Salamanca, Spain; 5 Faculty of Nursing and Physiotherapy, University of Salamanca, 37007 Salamanca, Spain; tvicente@usal.es; 6 Laboratory of Innovation in Nursing and Health Care, Biosanitary Institute of Salamanca (IBSAL), 37007 Salamanca, Spain; 7 Department of Neurosurgery, Salamanca University Assistance Complex (CAUSA), 37007 Salamanca, Spain; luistorres@usal.es

**Keywords:** microRNA, neurodevelopment, neurological disorders, neuropsychiatric disorders, biomarkers

## Abstract

MicroRNAs (miRNAs) are small non-coding RNAs that regulate gene expression at the post-transcriptional level and play important roles in the development and function of the nervous system. Through the coordinated regulation of multiple target genes and signalling pathways, miRNAs participate in key neurodevelopmental processes, including neural progenitor proliferation and differentiation, neuronal specification and migration, morphological maturation, synaptic development, and circuit formation. Their functions are strongly dependent on developmental stage, cell type, brain region, and expression level, and the extent of mechanistic characterization varies considerably among individual miRNAs. This review examines well-characterized and emerging neurodevelopment-related miRNAs, including members of the miR-9, miR-34, miR-107, miR-124, miR-132, miR-134, miR-137, miR-181, miR-185, miR-191, miR-200, miR-218, and miR-344 families, integrating evidence on their molecular mechanisms and functions during nervous system development. Their dysregulation is further considered in neurodevelopmental, neuropsychiatric, neurological, and neurodegenerative disorders, as well as in selected rare and metabolic or endocrine disorders with neurological involvement. We also examine the potential of circulating and tissue-associated miRNAs as diagnostic and prognostic biomarkers and the emerging therapeutic possibilities of miRNA modulation. Current evidence supports important roles for miRNAs in neurodevelopment and neurological disease but also highlights substantial differences between mechanistic and associative evidence. Methodological heterogeneity, differences between central and peripheral miRNA profiles, limited independent validation, and challenges associated with targeted delivery and off-target effects remain major barriers to their clinical translation.

## 1. Introduction

MicroRNAs (miRNAs) are small endogenous non-coding RNA molecules, generally 20–25 nucleotides in length, that act as critical post-transcriptional regulators of gene expression [1,2,3]. They function primarily by binding to complementary sequences within the 3′ untranslated region (3′UTR) of target messenger RNAs (mRNAs), leading to translational repression or mRNA degradation [2,4,5].

**Figure 1:**
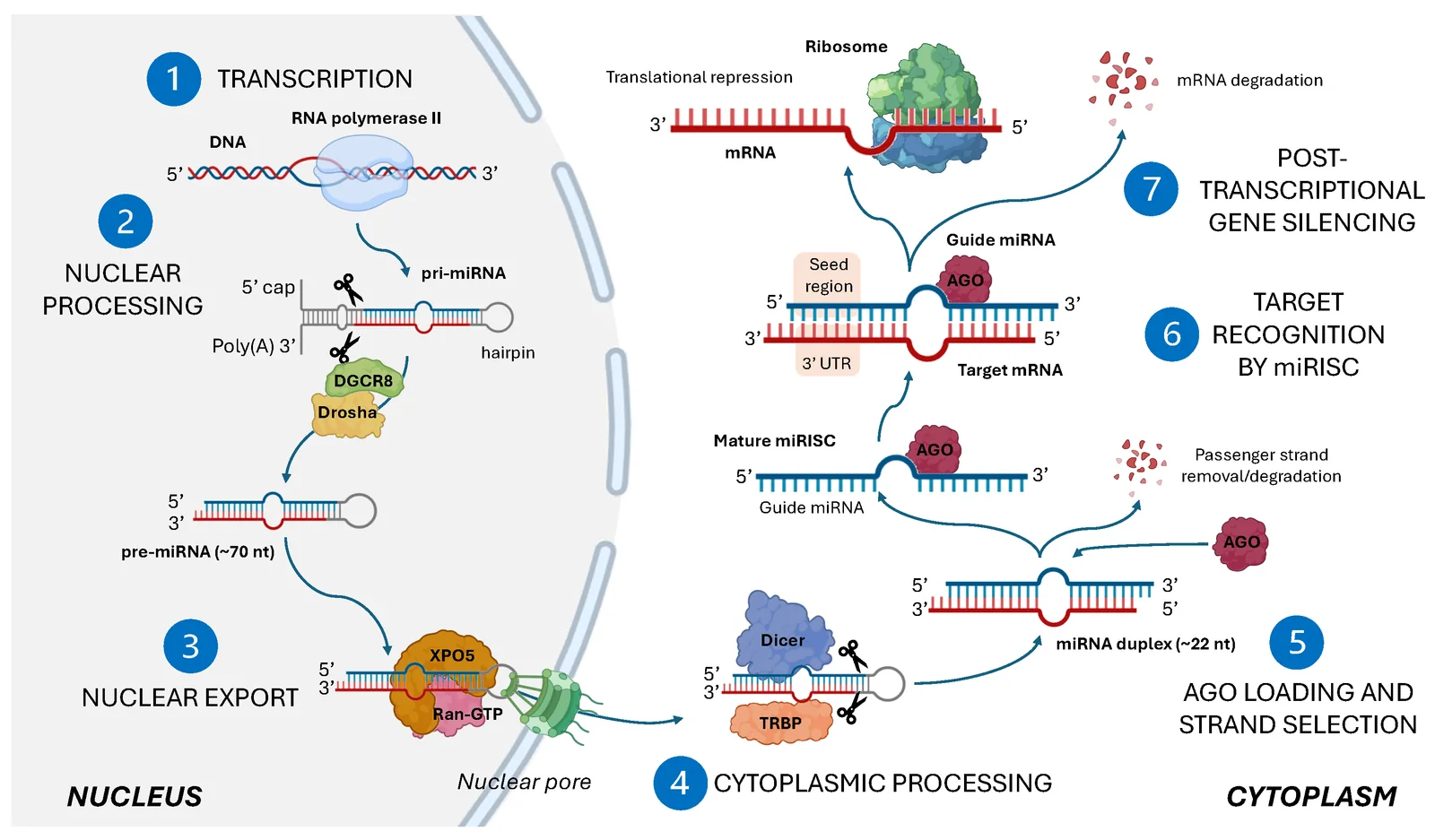
Canonical miRNA biogenesis and post-transcriptional gene silencing. AGO, Argonaute protein; DGCR8, DiGeorge syndrome critical region 8; DNA, deoxyribonucleic acid; miRNA, microRNA; miRISC, microRNA-induced silencing complex; mRNA, messenger RNA; nt, nucleotides; pre-miRNA, precursor microRNA; pri-miRNA, primary microRNA; Ran-GTP, GTP-bound Ras-related nuclear protein (Ran); RNA, ribonucleic acid; TRBP, trans-activation response RNA-binding protein; UTR, untranslated region; XPO5, Exportin-5. Figure partially created using BioRender. Garcia Barrado, M.J. (2026): https://BioRender.com/inyjmyg.

Although the miRNA biogenesis pathway has been extensively reviewed, its main steps are relevant for understanding their regulatory functions during neurodevelopment. As described in Figure 1 the canonical pathway begins in the nucleus, where RNA polymerase II transcribes miRNA genes into primary miRNA transcripts (pri-miRNAs), which contain characteristic hairpin structures, a 5′ cap, and a 3′ poly(A) tail [6]. Pri-miRNAs are recognized by the Microprocessor complex, composed of the RNase III endonuclease Drosha and the RNA-binding protein DGCR8. DGCR8 recognizes and positions the pri-miRNA, allowing Drosha-mediated cleavage and the release of an approximately 70-nucleotide precursor miRNA (pre-miRNA) with a stem–loop structure and a two-nucleotide 3′ overhang [7,8].

Pre-miRNAs are subsequently exported from the nucleus to the cytoplasm by Exportin-5 (XPO5) in a Ran-GTP-dependent process that also protects them from nucleolytic degradation [9]. In the cytoplasm, the RNase III endonuclease Dicer, together with associated proteins such as TRBP, cleaves the terminal loop of the pre-miRNA to generate an approximately 22-nucleotide miRNA duplex [10]. This duplex is then loaded onto an Argonaute (AGO) protein to form the RNA-induced silencing complex (RISC). One strand is retained as the guide strand, whereas the passenger strand is generally discarded. The guide miRNA directs RISC towards complementary sequences in target mRNAs, resulting predominantly in translational repression or mRNA degradation [11,12,13].

The fundamental importance of this machinery during nervous-system development is illustrated by the severe consequences of disrupting core components such as Dicer and DGCR8. Their complete loss can result in profound developmental abnormalities, including embryonic lethality, defective brain morphogenesis, and widespread neuronal death [2,5,14] (Figure 1).

Beyond their biogenesis, the biological relevance of miRNAs lies in their capacity to provide precise spatial and temporal control of gene expression during brain development [15,16]. Their expression is highly dynamic and spatiotemporally restricted, allowing individual miRNAs to function as both broad regulators and fine-tuners of gene networks controlling successive stages of neurodevelopment [14,17,18,19].

Different miRNAs participate in distinct but overlapping developmental processes. Neural induction and the establishment of progenitor pools involve, among others, the let-7, miR-200, and miR-96 families [14,20], whereas miR-9, miR-137, and the miR-17-92 cluster contribute to neural stem-cell proliferation and cell-fate determination [4,18,21]. Neuronal migration and positioning within the developing cortex and hippocampus are regulated by miRNAs including miR-9, miR-132, and miR-19 [14,18]. Other miRNAs, including miR-124, miR-134, and miR-218, participate in neuronal maturation, synaptogenesis, and synaptic plasticity [22,23]. During embryonic and postnatal development, these regulatory networks therefore contribute to neural stem-cell self-renewal, lineage specification, neuronal migration, axon guidance, dendritic morphogenesis, and ultimately the assembly and maturation of functional neuronal circuits [24,25].

The functional complexity of miRNA-mediated regulation arises partly from the ability of individual miRNAs to regulate multiple mRNA targets simultaneously, thereby generating extensive regulatory networks [2,17,26]. Conversely, several miRNAs may cooperate in the regulation of common developmental pathways. For example, miR-124 and miR-9 contribute to neuronal differentiation by suppressing antineurogenic regulatory mechanisms involving PTBP1 and REST [22,27,28]. miRNAs can also be organized into families and genomic clusters, such as the miR-182/183/96 cluster and the miR-34 family, whose coordinated and region-specific expression provides an additional level of regulation within the developing and mature nervous system [28,29].

An additional level of regulation occurs within neuronal subcellular compartments. Localization of miRNAs and components of the miRNA-processing machinery at dendrites and synapses enables local, activity-dependent control of mRNA translation, providing a mechanism through which miRNAs can influence synaptic maturation and plasticity [15]. Thus, miRNA function extends from the regulation of early cell-fate decisions to the local control of gene expression required for the maturation and adaptation of neuronal networks.

Given the dependence of brain development on tightly coordinated gene-expression programs, alterations in miRNA expression or function can disrupt the formation and maintenance of neuronal circuits. Genetic variants, epigenetic alterations, and environmental factors affecting miRNA regulation have consequently been associated with a broad spectrum of neurodevelopmental, neuropsychiatric, and neurological disorders, including autism spectrum disorder, schizophrenia, bipolar disorder, epilepsy, and neurodegenerative diseases [23,26,28,30]. These associations have also stimulated growing interest in miRNAs as potential molecular biomarkers and therapeutic targets.

In this context, this review focuses on a selected group of miRNAs involved in key processes of nervous system development, including neural progenitor regulation, neuronal differentiation, migration, neurite and dendritic development, synaptogenesis, and neuronal plasticity [22,27]. The selected miRNAs represent different levels of current knowledge and can be broadly divided into two groups: well-characterized miRNAs, for which substantial experimental and mechanistic evidence supports their involvement in neurodevelopment, and emerging miRNAs, whose roles in neural development have begun to be identified but for which the underlying molecular mechanisms and functional relevance remain less extensively characterized. By considering these different levels of evidence, this review aims not only to summarize the physiological functions of these miRNAs during nervous system development, but also to examine how their dysregulation may be associated with neurological, neuropsychiatric, and neurodegenerative disorders [27,28,30].

## 2. Literature Search and Selection Strategy

An initial exploratory search was conducted using OpenEvidence to identify miRNAs potentially relevant to neurodevelopment and nervous system disorders. This preliminary mapping was used to define a set of candidate miRNAs for further investigation. Each candidate miRNA was subsequently examined through targeted searches in PubMed, combining the individual miRNA name with terms related to neurodevelopment and neurological disease (e.g., “miR-9 AND neurodevelopment”, “miR-124 AND neurodevelopment”, or “miRNA AND neurological disease”). Additional research was conducted using disease-specific terms when relevant, and the reference lists of selected articles were examined to identify additional pertinent studies.

miRNAs were prioritized based on their reported involvement in key neurodevelopmental processes and the availability of experimental or mechanistic evidence supporting their biological role. Particular attention was given to miRNAs involved in neural progenitor regulation, neuronal differentiation and migration, neurite and dendritic development, synaptogenesis, and neuronal plasticity. The selection intentionally included both well-characterized miRNAs with established molecular mechanisms and emerging miRNAs for which evidence was more limited but suggested a potentially relevant role in neurodevelopment. Disease areas were subsequently selected according to the evidence linking these miRNAs or related miRNA regulatory mechanisms to nervous system pathology, with emphasis on neurodevelopmental, neuropsychiatric, neurological, and neurodegenerative disorders. Rare diseases and metabolic or endocrine disorders were included selectively when they provided relevant mechanistic links between miRNA dysregulation and nervous system development or function.

No publication date restrictions were applied, allowing the inclusion of both seminal mechanistic studies and recent evidence. The literature search was updated through August 2026.

## 3. MicroRNAs Involved in Neurodevelopment

Among the miRNAs involved in neurodevelopment, miR-9, miR-34, miR-107, miR-124, miR-132, miR-134, miR-137, miR-181, miR-185, miR-191, miR-200, miR-200a, miR-218, miR-344b, and miR-344c have been associated with different aspects of neurogenesis and neuronal maturation. However, the available evidence differs substantially among them. Whereas miRNAs such as miR-9 and miR-124 have extensively characterized regulatory networks involving multiple molecular targets and developmental processes, others, particularly miR-344b and miR-344c, remain comparatively poorly understood. The following sections therefore examine each miRNA according to the available evidence, focusing on its expression and regulation, molecular targets, and functional contribution to neurodevelopment.

### 3.1. MicroRNA miR-9

#### 3.1.1. miR-9: Expression and Regulatory Mechanisms

MicroRNA-9 (miR-9) is one of the most evolutionarily conserved and abundant miRNAs in the developing and adult vertebrate brain and plays a central role in neurogenesis, neuronal differentiation, progenitor-cell maintenance, and neuronal maturation [25,31,32]. In mammals, miR-9 is encoded by three genomic loci, *MIR9-1*, *MIR9-2*, and *MIR9-3*, which generate an identical mature sequence. In zebrafish, genome duplication has expanded this family to seven paralogues [31,33,34] (Table 1).

During zebrafish neurodevelopment, miR-9 expression is highly dynamic, initially emerging in the telencephalon and subsequently extending towards the midbrain, hindbrain, and spinal cord [25,31,38]. During neurogenesis, it is predominantly expressed in the ventricular zone, where neural progenitor cells reside, although it is also detected in specific populations of differentiated neurons [25,38]. Conversely, miR-9 is excluded from non-neurogenic boundary regions such as the midbrain–hindbrain boundary, where its absence contributes to progenitor maintenance [14,25,38].

The expression of miR-9 is controlled by an interconnected transcriptional network. Cell-based studies have shown that Mef2C directly activates miR-9 transcription by binding to the *miR-9-2* promoter, while miR-9 represses HDAC4, a negative regulator of Mef2C, establishing a positive feedback mechanism that sustains miR-9 expression during neuronal differentiation [33]. CREB also promotes miR-9 transcription in response to neuronal differentiation signals. In parallel, removal of REST-mediated repression and subsequent targeting of REST mRNA by miR-9 establish a double-negative feedback loop that reinforces neuronal differentiation [25,34].

Conversely, studies using zebrafish, mouse, and cell-based models indicate that TLX/Nr2e1 and the Notch effector Hes1 participate in regulatory networks that repress miR-9 transcription and contribute to the maintenance and self-renewal of neural progenitors [25,33,36]. In mouse models, miR-9 activity is additionally modulated post-transcriptionally by RNA-binding proteins, including Elavl2, Elavl1, and Musashi1, which can modify its effects on targets such as FoxG1 and Nr2e1 [35].

#### 3.1.2. Functional Role in Neurodevelopment

The broad range of miR-9 targets indicates that its function extends beyond the regulation of individual transcription factors. By simultaneously modulating transcriptional regulators, cytoskeletal proteins, and chromatin-remodelling factors, miR-9 coordinates the transition from neural progenitor maintenance to neuronal differentiation and maturation [14,25,33]. In human neural progenitor cells, its activity is also associated with networks involved in plasma-membrane organization, cell morphogenesis, and cell adhesion [39].

Overall, miR-9 represents a central regulatory hub connecting transcriptional, post-transcriptional, cytoskeletal, and epigenetic mechanisms, allowing the precise temporal coordination of progenitor maintenance, neuronal differentiation, and subsequent neuronal maturation [25,31].

### 3.2. MicroRNA miR-34

#### 3.2.1. miR-34: Expression and Regulatory Mechanisms

The miR-34 family is an evolutionarily conserved group comprising three mature members, miR-34a, miR-34b, and miR-34c [40,41,42,43]. These members are encoded by two transcriptional units: *MIR34A* is located on chromosome 1, whereas *MIR34B* and *MIR34C* are co-transcribed from a cluster on chromosome 11 in humans [40,41,44,45].

In the mammalian brain, miR-34a is broadly expressed, with particularly high levels in the cerebral cortex and hippocampus, whereas miR-34b and miR-34c display a more restricted distribution [40,43,46,47]. In rodent models, miR-34a is detected in progenitor niches during cortical neurogenesis and progressively increases during neuronal maturation [42,44]. In mouse models, the miR-34 family also interacts functionally with the miR-449 cluster, with their coordinated activity contributing to normal ventricular/subventricular development and ciliogenesis [48,49].

Cell-based studies have identified p53 as a major transcriptional regulator of miR-34, directly activating miR-34a in response to differentiation signals such as NGF [40,44,46]. TAp73 also promotes miR-34a expression during neuronal commitment [40,46]. miR-34a participates in feedback regulation with p53, while Notch signalling and epigenetic mechanisms, including promoter methylation, provide additional regulatory levels [40,41,44] (Table 2).

#### 3.2.2. Functional Role in Neurodevelopment

Evidence from rodent and cell-based models indicates that the miR-34 family contributes to the transition from neural progenitors to postmitotic neurons by promoting cell-cycle exit and acquisition of a differentiated neuronal state [40,44,45]. However, its effects are highly dependent on developmental stage and expression level. Appropriate miR-34a activity contributes to neurite extension and neuronal maturation, whereas excessive expression can impair differentiation, reduce synaptic proteins, and decrease dendritic complexity [42,43,47,50].

Thus, miR-34a appears to function primarily as a fine regulator of neuronal maturation rather than simply as an on/off inducer of differentiation.

### 3.3. MicroRNA miR-107

#### 3.3.1. miR-107: Expression and Regulatory Mechanisms

miR-107 is a brain-enriched member of the conserved miR-103/107 family [27]. Its expression during central nervous system development is dynamic and spatially restricted. In the developing zebrafish hindbrain, miR-107 is initially detected in proliferating radial glial progenitors and subsequently shifts towards periventricular and postmitotic regions, accompanying the transition from progenitor states to newly differentiated neurons [51]. In mammalian models, its expression progressively increases from early embryonic development to birth [19,52].

Unlike miR-9 or miR-34, specific transcription factors directly controlling mammalian miR-107 remain poorly characterized. Its regulation appears to involve important post-transcriptional mechanisms [14,52]. Particularly relevant is the direct targeting of the Dicer mRNA, establishing a regulatory interaction through which miR-107 can influence the biogenesis of other miRNAs [51]. Its expression is also sensitive to environmental and pharmacological stimuli, as illustrated by increased miR-107 following ketamine exposure in embryonic stem cell-derived neurons [53] (Table 3).

#### 3.3.2. Functional Role in Neurodevelopment

In zebrafish, miR-107 contributes to the balance between progenitor proliferation and neuronal differentiation through the miR-107–Dicer–miR-9 axis. miR-107-mediated Dicer destabilization contributes to changes in mature miR-9 as progenitor cells leave the ventricular zone and differentiate, thereby helping establish an appropriate temporal balance between progenitor maintenance and neuronal production [51]. Beyond this developmental axis, miR-107 may also influence neuronal survival and neurite development through regulation of BDNF under specific experimental conditions [53].

### 3.4. MicroRNA miR-124

#### 3.4.1. miR-124: Expression and Regulatory Mechanisms

miR-124 is an evolutionarily ancient and highly conserved neuronal miRNA and represents one of the most abundant miRNAs in the vertebrate central nervous system [22,54,55,56]. In humans and mice, it is encoded by three independent genomic loci, *MIR124-1*, *MIR124-2*, and *MIR124-3* [22,54,56,57].

Its expression is closely associated with neuronal differentiation. miR-124 is low or absent in neural stem and glial cells but is strongly induced as progenitors transition towards postmitotic neurons, with expression progressively increasing throughout brain development and maturation [22,23,56,58,59].

Cell-based studies have identified REST/NRSF-mediated repression as a major regulatory mechanism controlling miR-124 expression in non-neuronal cells. During neurogenesis, loss of REST-mediated repression permits miR-124 expression, while miR-124 in turn inhibits components of the REST pathway, reinforcing neuronal identity [22,58,60]. PTBP1 provides an additional level of regulation by interfering with the processing of pri-miR-124-1 and inhibiting production of mature miR-124 in embryonic stem cells [56,61] (Table 4).

#### 3.4.2. Functional Role in Neurodevelopment

Studies in mouse and cell-based models have shown that one of the best-characterized mechanisms of miR-124 involves repression of PTBP1, which promotes a transition towards neuron-specific alternative splicing and PTBP2 expression [22,58,63]. Evidence from different vertebrate and cell-based models further indicates that miR-124-mediated repression of SCP1, SOX9, and components of anti-neural signalling pathways suppresses alternative cellular fates and reinforces neuronal differentiation [22,56,62,64].

Cell-based studies have also implicated miR-124 in cell-cycle exit through the regulation of CDK6 and other proliferation-associated genes. In parallel, evidence from experimental models supports a role for miR-124 in neuronal migration, axonal extension, and dendritic morphogenesis through the regulation of cytoskeletal pathways involving ROCK1, RhoG, and Cdc42 [22,56,66,67]. However, complete deletion of all miR-124 loci in human induced pluripotent stem cells did not prevent neuronal differentiation, although it resulted in altered neuronal morphology, neurite outgrowth, neurotransmitter specification, and long-term viability, indicating that miR-124 may refine neuronal identity and maturation rather than being strictly required for the initiation of neurogenesis [67].

Its function extends into mature neuronal physiology, where miR-124 contributes to synaptic plasticity and long-term memory through pathways involving CREB and PTPN1 [23,54,56]. Taken together, these mechanisms support its classification as one of the principal molecular regulators of neuronal identity and maturation.

### 3.5. MicroRNA miR-132

#### 3.5.1. miR-132: Expression and Regulatory Mechanisms

miR-132 is a highly conserved, brain-enriched member of the miR-212/132 family. Both miRNAs are processed from the same polycistronic primary transcript and share an identical seed sequence, although miR-132 is the predominant functional member of this cluster in the CNS [68,69,70].

Across human, non-human primate, and mouse brain development, miR-132 expression shows a developmentally regulated pattern, increasing during neuronal maturation and periods associated with migration and synaptogenesis. In the adult brain, it remains highly expressed in regions including the cortex, hippocampus, and suprachiasmatic nucleus [69,71].

Studies using cell-based models and mouse neuronal cultures have shown that miR-132 transcription is predominantly controlled by CREB and is strongly induced by neuronal activity, synaptic stimulation, and BDNF through MAPK/ERK1/2 and MSK1 signalling [69,70,72,73]. Conversely, REST can repress the miR-212/132 promoter [70,72] (Table 5).

#### 3.5.2. Functional Role in Neurodevelopment

miR-132 coordinates neuronal migration, dendritic growth, spinogenesis, axonal development, and synaptic plasticity. Evidence from experimental models indicates that appropriate miR-132 expression contributes to neuronal migration and the integration of newly generated neurons during cortical development [72,74,76,77].

Its activity-dependent regulation also makes miR-132 particularly important during later neuronal maturation. In mouse neuronal cultures, miR-132 promotes dendritic growth and spine formation through repression of p250GAP and modulation of Rac1-PAK signalling [69,71,78]. miR-132 is additionally involved in experience-dependent plasticity in the visual cortex and maintenance of hippocampal synaptic strength [68,73,78,79].

### 3.6. MicroRNA miR-134

#### 3.6.1. miR-134: Expression and Regulatory Mechanisms

miR-134 is a brain-enriched miRNA located within the large miR-379–410 cluster in the imprinted *Dlk1*-*Gtl2* domain [14,80,81]. Its expression increases during brain development and reaches high levels during periods of synaptic maturation [80].

Cell-based studies have shown that miR-134 is enriched in dendritic and synaptic compartments, where its local processing and activity contribute to the regulation of protein synthesis. Pre-miR-134 is transported to synaptic regions by the RNA-binding protein DHX36 and subsequently processed by Dicer [82].

Transcription of the cluster is controlled by epigenetic regulation of the Dlk1-Gtl2 locus. In cell-based models, miR-134 expression is regulated by neuronal activity-related signalling, with Mef2 promoting its expression and BDNF contributing to its induction during dendritic maturation [80,82,83]. In astrocytes, bFGF induces miR-134 through ERK and PI3K/Akt signalling [82] (Table 6).

#### 3.6.2. Functional Role in Neurodevelopment

miR-134 displays strongly stage-dependent functions. In cell-based models of early differentiation, miR-134 promotes ectodermal and neuroectodermal differentiation by repressing pluripotency-associated factors [81]. Studies using cell-based models and rodent embryos indicate that miR-134 contributes to cortical progenitor proliferation and survival and regulates neuronal migration through repression of Dcx, thereby influencing radial migration and cortical positioning [14,84].

During neuronal maturation, cell-based studies indicate that miR-134 regulates dendritic and synaptic morphology through targets including LimK1 and Pum2, providing a mechanism by which neuronal activity can influence dendritic development and spine structure [80,83]. Thus, miR-134 illustrates particularly well the developmental-stage dependence of miRNA function.

### 3.7. MicroRNA miR-137

#### 3.7.1. miR-137: Expression and Regulatory Mechanisms

miR-137 is a brain-enriched miRNA involved in the regulation of neural stem-cell proliferation, neuronal differentiation, and maturation. Studies in mouse neural stem cells have shown that increased miR-137 expression reduces progenitor proliferation and promotes neuronal differentiation. A regulatory loop involving miR-137, the nuclear receptor TLX, and the histone demethylase LSD1 contributes to the coordinated transition between neural stem-cell proliferation and differentiation [85]. miR-137 is also connected to epigenetic regulation through its interaction with the histone methyltransferase EZH2, thereby modulating adult hippocampal neurogenesis [86]. In addition to these effects on progenitor regulation, miR-137 influences later stages of neuronal development, with increased miR-137 activity impairing neuronal maturation and dendritic morphogenesis through repression of the ubiquitin ligase Mib1 [87] (Table 7).

#### 3.7.2. Functional Role in Neurodevelopment

miR-137 exerts stage-dependent functions during neurogenesis, acting at both progenitor and neuronal maturation stages. In mouse neural stem cells, increased miR-137 expression reduces proliferation and promotes neuronal differentiation [85], while its regulation of EZH2 contributes to adult hippocampal neurogenesis [86]. At later stages, increased miR-137 activity can impair neuronal maturation and dendritic development through regulation of Mib1 [87]. Together, these findings indicate that the effects of miR-137 are strongly dependent on developmental context and expression level, allowing it to coordinate the transition from proliferative neural progenitors to morphologically mature neurons [3,14].

### 3.8. MicroRNA miR-181

#### 3.8.1. miR-181: Expression and Regulatory Mechanisms

The miR-181 family acts as a post-transcriptional regulator of neuronal development and maturation. Studies using primary neuronal cultures from rat embryos have shown that miR-181 contributes to neuronal structural development, including dendritic arborization, synapse formation, and axonal growth [88].

Regulation of miR-181 expression has also been linked to signalling pathways involved in neural development. During neonatal cerebellar development, α1ACT induces the expression of miR-181 family members, particularly miR-181a-5p, influencing pathways associated with neurogenesis, neuronal differentiation, and cell survival [89]. In addition, studies in Lewis rats, primary hippocampal neurons, and cell-based models have identified an IGF-1–miR-181a–CREB1 regulatory axis. IGF-1 decreases miR-181a expression, whereas miR-181a directly targets the 3′UTR of *Creb1* and negatively regulates its expression, thereby influencing dendritic growth [90] (Table 8).

#### 3.8.2. Functional Role in Neurodevelopment

miR-181 contributes to the structural maturation of developing neurons. Studies using primary neuronal cultures indicate that members of the miR-181 family regulate dendritic arborization, synapse formation, and axonal growth [88]. In primary hippocampal neurons from newborn rats, miR-181a negatively regulates dendritic growth, as its overexpression reduces dendritic length and branching, whereas its inhibition produces the opposite effect [90]. Together, these findings support a role for miR-181 family members in the establishment and maturation of neuronal connectivity.

### 3.9. MicroRNA miR-185

#### 3.9.1. miR-185: Expression and Regulatory Mechanisms

miR-185 participates in neural progenitor proliferation and differentiation. Studies using mouse embryonic neural progenitor cells and mouse embryos have shown that miR-185 expression increases during neural progenitor differentiation and promotes glial differentiation while restricting progenitor proliferation. Accordingly, inhibition of miR-185 expands the progenitor population and reduces gliogenesis [91].

A particularly relevant feature of miR-185 is its localization within the 22q11.2 region. In mouse models of 22q11.2 deletion, reduced miR-185 expression has been associated with alterations in neuronal development and morphology [92,93].

The transcriptional mechanisms controlling miR-185 expression during mammalian neurodevelopment remain poorly characterized (Table 9).

#### 3.9.2. Functional Role in Neurodevelopment

miR-185 contributes to the balance between progenitor proliferation and glial differentiation and also influences neuronal dendritic and spine development. Its reduction alters neuronal morphogenesis and hippocampal structure, illustrating how miRNA dosage can influence both cellular differentiation and later neural architecture [91,92,93].

### 3.10. MicroRNA miR-191

#### 3.10.1. miR-191: Expression and Regulatory Mechanisms

miR-191 displays regulated expression during neural differentiation and neuronal maturation. In mouse P19 embryonic carcinoma cells undergoing neural differentiation, miR-191 expression increases markedly following the acquisition of a neural progenitor phenotype [94]. In primary hippocampal neuronal cultures, miR-191 expression is also modulated in response to NMDA receptor-dependent neuronal activity, suggesting that its regulation extends to later stages of neuronal maturation and activity-dependent plasticity [95]. However, the transcriptional mechanisms controlling miR-191 expression during neurodevelopment remain poorly characterized (Table 10).

#### 3.10.2. Functional Role in Neurodevelopment

Current evidence supports an association of miR-191 with neural differentiation and neuronal maturation, although its specific functions during neurodevelopment remain incompletely characterized. Functional studies in neuronal cell models have shown that miR-191-5p can promote neurite outgrowth and have identified DAPK1 as a direct molecular target [96]. However, these experiments were performed primarily in the context of Alzheimer’s disease-related mechanisms, and whether the miR-191-5p–DAPK1 axis contributes to physiological neurodevelopment remains to be established.

### 3.11. MicroRNA miR-200 Family

#### 3.11.1. miR-200 Family: Expression and Regulatory Mechanisms

The miR-200 family comprises miR-200a, miR-200b, miR-200c, miR-141, and miR-429, whose expression is dynamically regulated during neuronal differentiation and maturation. Studies using mouse embryos, primary neural progenitor cultures, PC12 cells, and neural stem cells have shown increased miR-200 family expression during neuronal differentiation. Mechanistically, miR-200 promotes cell-cycle exit and neuronal differentiation through repression of SOX2 and E2F3, which in turn regulate miR-200 expression, establishing a negative feedback loop between miR-200 and factors involved in progenitor maintenance [97]. Consistent with this mechanism, miR-200 overexpression promotes neurite formation and neuronal differentiation, whereas its inhibition favours cell proliferation. Additional evidence indicates that these effects are associated with reduced expression of stemness-related transcription factors, including SOX2 and KLF4 [98].

At later stages of neurogenesis, in vivo studies of postnatal olfactory bulb neurogenesis in mice have shown that miR-200 family expression increases during neuronal maturation. Gain- and loss-of-function experiments identified Zeb2 as a functional target of the miR-200 family, linking its developmental regulation to late neuronal differentiation and maturation [99] (Table 11).

#### 3.11.2. Functional Role in Neurodevelopment

The miR-200 family appears to regulate neuronal development in a stage-dependent manner. During early neurogenesis, miR-200 family members contribute to the transition from proliferative neural progenitors towards neuronal differentiation through regulatory interactions involving Sox2 and E2F3 [97]. Consistent with this function, increased miR-200 activity promotes neuronal differentiation and neurite formation in cellular models [98]. At later stages, studies of postnatal olfactory bulb neurogenesis indicate that miR-200 family members contribute to neuronal differentiation and maturation through repression of Zeb2 [99]. Together, these findings suggest that the miR-200 family participates at successive stages of neurogenesis, from progenitor cell-cycle regulation to neuronal maturation.

### 3.12. MicroRNA miR-218

#### 3.12.1. miR-218: Expression and Regulatory Mechanisms

miR-218 displays cell-type- and region-specific expression during nervous system development. In the developing mouse cortex, miR-218 is enriched in layer V projection neurons, where its expression is developmentally regulated. Experimental overexpression disrupts radial migration, neuronal morphogenesis, and cortical distribution through repression of Satb2 [100]. In the developing spinal cord, miR-218 is directly integrated into the transcriptional programme specifying motor neuron identity. The Isl1–Lhx3 complex directly induces miR-218 expression, which in turn represses genes associated with interneuron and neural progenitor programmes, thereby promoting motor neuron specification [101].

miR-218 is also developmentally regulated during the assembly and maturation of neuronal circuits. In the mouse hippocampus, its expression increases during early postnatal development and is enriched in both excitatory principal neurons and GABAergic interneurons, where it contributes to the establishment of stable neuronal networks [102]. In the dopaminergic system, studies using mouse and cell-based models have shown that miR-218 promotes dopaminergic differentiation and contributes to neuronal functional maturation through the regulation of synaptic gene networks involved in excitability and neurotransmitter release [103] (Table 12).

#### 3.12.2. Functional Role in Neurodevelopment

miR-218 illustrates the strong cell-type and developmental-stage specificity of miRNA function. In the developing cortex, appropriate miR-218 expression is required for normal neuronal migration and morphogenesis, whereas in the spinal cord it acts downstream of Isl1–Lhx3 to establish motor neuron identity [100,101]. At later stages of neuronal development, miR-218 contributes to circuit assembly and functional maturation. Early postnatal inhibition in the mouse hippocampus disrupts depolarizing GABAergic signalling, dendritic-spine morphology, intrinsic excitability, and long-term network stability [102]. In dopaminergic neurons, miR-218 additionally promotes differentiation and regulates synaptic gene networks involved in excitability and dopamine release [103]. Together, these findings indicate that miR-218 functions across successive levels of neurodevelopment, from neuronal subtype specification and migration to synaptic and circuit maturation.

### 3.13. MicroRNA miR-344 Family

#### 3.13.1. miR-344 Family: Expression and Regulatory Mechanisms

The miR-344 family comprises multiple miRNAs encoded within a cluster on mouse chromosome 7 and displays developmentally regulated expression in the nervous system. miR-344-3p is enriched in neural tissues during mouse embryonic development, including the cerebral cortex, midbrain, hindbrain, cerebellum, spinal cord, and dorsal root ganglia, with expression decreasing markedly after birth [104]. miR-344b and miR-344c also show distinct spatiotemporal expression patterns during mouse brain development and are detected in the nuclei of immature neurons. While miR-344b becomes undetectable by in situ hybridization postnatally, miR-344c persists in selected adult brain regions, including the olfactory bulb and cerebellum [105].

The molecular mechanisms of miR-344 family members during neurodevelopment remain poorly characterized. *Olig2* and *Otx2* were predicted as potential targets of miR-344b and miR-344c, respectively, but direct regulation was not supported experimentally [105].

#### 3.13.2. Functional Role in Neurodevelopment

The developmental expression of miR-344 family members suggests a potential role in early neurodevelopmental processes [104,105]. However, direct evidence demonstrating specific functions in neural progenitor proliferation, neuronal differentiation, or maturation remains limited. Thus, the miR-344 family should currently be considered an emerging group of developmentally regulated miRNAs whose molecular targets and specific neurodevelopmental functions remain to be established.

## 4. Integrated Role of miRNAs in Neurodevelopment

Taken together, the miRNAs discussed above form a complex regulatory network operating across successive and overlapping stages of nervous system development. During early neurogenesis, miRNAs contribute to the balance between neural progenitor maintenance, cell-cycle exit, and neuronal or glial differentiation by regulating transcription factors, epigenetic regulators, RNA-processing proteins, and signalling pathways. At subsequent stages, they participate in neuronal subtype specification, migration, neurite and dendritic development, and morphological maturation. Finally, several miRNAs contribute to synapse formation, neuronal excitability, activity-dependent plasticity, and the assembly of functional neural circuits [14,106].

These functions are highly dependent on developmental stage, cellular context, and miRNA expression level. Some miRNAs, such as miR-9 and miR-124, act across multiple stages of neurogenesis, whereas others show more restricted cell-type- or stage-specific functions. miR-218, for example, illustrates how a single miRNA can participate in distinct developmental programmes depending on neuronal identity, from motor neuron specification to cortical development and circuit maturation. Conversely, emerging miRNAs such as members of the miR-344 family are characterized primarily by developmentally regulated expression, while their molecular targets and functional roles remain insufficiently defined.

Overall, miRNAs should therefore not be considered isolated regulators of individual genes, but components of interconnected regulatory networks that provide temporal, spatial, and quantitative control of neurodevelopment. Their capacity to regulate multiple molecular targets and pathways enables coordinated transitions from proliferative neural progenitors to differentiated and functionally integrated neurons. Consequently, alterations in miRNA expression or activity may disrupt several developmental processes simultaneously, providing a potential mechanistic link between miRNA dysregulation and nervous system disorders [14,23,27].

While the preceding sections focused on selected miRNAs with established or emerging roles in neurodevelopment, the following sections adopt a broader perspective to examine miRNA dysregulation across neurological, neurodevelopmental, neuropsychiatric, and neurodegenerative disorders. Accordingly, additional miRNAs identified in disease-specific studies are also considered, particularly where they provide relevant mechanistic, diagnostic, prognostic, or therapeutic information.

### 4.1. Relationship Between microRNAs and Neurological, Neuropsychiatric and Neurocognitive Disorders

Dysregulation of miRNA expression has been reported across a broad range of neurological, neurodevelopmental, and neuropsychiatric disorders, including autism spectrum disorder, intellectual disability, schizophrenia, epilepsy, and neurodegenerative diseases [3,23,28,107,108,109]. Altered miRNA activity may affect molecular pathways involved in neural progenitor differentiation, neuronal migration, synaptic development and plasticity, neuronal excitability, inflammation, and cellular homeostasis, thereby linking miRNA regulation to processes relevant to nervous system disease.

Importantly, the biological significance of miRNA dysregulation depends on the type of evidence available. Experimental manipulation in cellular and animal models can provide mechanistic evidence linking individual miRNAs to specific molecular or cellular phenotypes, whereas altered miRNA expression in human brain tissue or peripheral samples primarily establishes an association with disease. Such alterations may contribute to disease mechanisms, but they may also arise as secondary or compensatory responses to pathological processes. Therefore, evidence from experimental models, human tissues, and circulating biomarkers should be interpreted according to their respective biological and clinical contexts.

### 4.2. Neurodevelopmental and Neuropsychiatric Disorders

Altered miRNA expression has been reported across several neurodevelopmental and neuropsychiatric disorders, including autism spectrum disorder, schizophrenia, and affective disorders, in both brain tissue and peripheral samples [107,109,110,111]. In addition, genetic variation affecting neurodevelopment-related miRNAs has been associated with susceptibility to psychiatric disorders, providing an additional link between miRNA regulation, neurodevelopment, and psychiatric disease [112]. Many disease-associated miRNAs regulate molecular pathways involved in neuronal differentiation, circuit development, synaptic function, and plasticity [14,107,109]. However, the biological significance of altered miRNA expression depends on the tissue examined and the type of evidence available, and these changes may represent either contributors to disease mechanisms or secondary responses to pathological processes.

### 4.3. Autism Spectrum Disorder

Altered miRNA expression has been reported in autism spectrum disorder (ASD) across different biological samples, including post-mortem brain tissue, blood-derived samples, and saliva [113,114,115,116,117]. Despite substantial heterogeneity across studies and biological samples, miR-106b-5p, miR-155-5p, and miR-146a-5p have been among the most consistently reported as upregulated in ASD, whereas miR-328-3p has been consistently downregulated [116].

Functional and bioinformatic analyses suggest that ASD-associated miRNAs may regulate genes and pathways involved in neurodevelopment, synaptic function, cell signalling, adhesion, metabolism, and immune regulation [114,115,117,118]. Nevertheless, the functional significance of individual miRNAs varies according to the available evidence, as many associations are based on differential expression and target-prediction analyses rather than direct experimental demonstration. Experimental evidence from animal models provides additional support for the involvement of miRNA dysregulation in ASD-related phenotypes. For example, reduced expression of miR-19a-3p, miR-361-5p, miR-3613-3p, miR-150-5p, miR-126-3p, and miR-499a-5p was detected in the serum of children with ASD, with related expression changes also observed in blood and brain tissue from valproic acid-induced and *Cc2d1a*^+/−^ mouse models of ASD [119].

Circulating and salivary miRNAs have also been extensively investigated as potential non-invasive biomarkers for ASD. Differential expression profiles have been reported in serum, plasma, and saliva, and several multi-miRNA signatures have been proposed for distinguishing children with ASD from typically developing controls [120,121,122,123,124]. In serum, a combined signature of miR-197-5p and miR-500a-5p showed high diagnostic performance in a cohort of children with ASD and typically developing controls [123]. More recently, analysis of plasma from children aged 2-4 years identified a four-miRNA panel comprising miR-4433b-5p, miR-15a-5p, miR-335-5p, and miR-1180-3p with an area under the ROC curve of 0.936; several miRNAs also correlated with age, highlighting developmental age as an important variable in the interpretation of circulating miRNA profiles [124].

### 4.4. Schizophrenia and Affective Disorders

In schizophrenia spectrum disorders (SSDs), miR-181b-5p and miR-34a-5p are among the most consistently dysregulated miRNAs, with increased expression reported in both post-mortem brain tissue and peripheral blood. These findings suggest the involvement of molecular pathways related to neurodevelopment, apoptosis, and cell survival [110]. Experimental evidence also supports persistent miRNA dysregulation following neurodevelopmental alterations. In a mouse model based on perinatal NMDA receptor hypofunction induced by ketamine, miR-451a was upregulated and miR-137-3p was downregulated in the adult prefrontal cortex, while miR-132-3p showed sex-dependent alterations [26].

A large human study of circulating miRNAs further identified both shared and disorder-specific alterations across psychotic and affective disorders. Analysis of the PsyCourse cohort revealed differential expression of multiple miRNAs, including miR-584-3p and miR-99b-5p, across psychotic disorders, affective disorders, and neurotypic controls, supporting the existence of both overlapping and distinct miRNA-related molecular profiles across severe psychiatric disorders [111].

### 4.5. Epilepsy and Disorders of Neuronal Excitability

miRNAs are increasingly recognized as regulators of molecular processes involved in epilepsy, including neuronal survival, synaptic function, neuroinflammation, and neuronal excitability. Changes in miRNA expression have been identified in hippocampal tissue from patients with temporal lobe epilepsy and in neural tissue from experimental models, supporting their involvement in the molecular alterations associated with epileptogenesis and recurrent seizures [28,125].

Among epilepsy-associated miRNAs, miR-134 has been particularly well characterized. Increased miR-134 expression has been reported in resected brain tissue from patients with temporal lobe epilepsy and across several experimental epilepsy models. As a synaptically enriched miRNA involved in the regulation of dendritic spine morphology and network excitability, its dysregulation may contribute to alterations in neuronal excitability associated with epilepsy [80,125]. Importantly, experimental inhibition of miR-134 using antisense oligonucleotides has produced antiseizure effects in multiple rodent models, increasing seizure thresholds, attenuating status epilepticus, and reducing the occurrence of spontaneous recurrent seizures after status epilepticus [80,125].

### 4.6. Neurodegenerative Diseases and Cognitive Impairment

miRNA dysregulation has been described across several neurodegenerative diseases, including Alzheimer’s disease (AD), Parkinson’s disease (PD), amyotrophic lateral sclerosis (ALS), Huntington’s disease (HD), and multiple sclerosis (MS). Despite their distinct etiologies, these disorders share alterations in miRNAs involved in neuronal and immune processes, with recurrently dysregulated miRNAs including miR-9-5p, miR-21-5p, members of the miR-29 family, miR-132-3p, miR-124-3p, miR-146a-5p, miR-155-5p, and miR-223-3p [126,127,128].

In AD and mild cognitive impairment (MCI), extensive changes in miRNA expression have been identified in post-mortem brain tissue, blood, and cerebrospinal fluid, although the direction and magnitude of these alterations vary according to the biological sample and disease stage [129,130]. Recent analyses of human brain tissue further support an association between miRNA dysregulation and cognitive decline and AD neuropathology. In the posterior cingulate cortex, distinct miRNA profiles have been identified across cognitively normal individuals, MCI, and AD, with specific miRNA networks associated with cognitive performance and neuropathological burden [131]. Similarly, analysis of the dorsolateral prefrontal cortex has identified numerous miRNAs associated with cognitive trajectory, amyloid and tau pathology, and clinical AD, with downstream pathways involving postsynaptic signalling, cellular senescence, and transcriptional regulation [132].

Changes in miRNA expression are also observed during earlier stages of cognitive impairment. Circulating and brain-derived miRNA profiles have been associated with cognitive performance and structural brain changes, including cortical thickness and hippocampal volume [133]. Plasma exosomal miRNAs also show stage-dependent changes across the normal cognition–MCI–AD continuum, suggesting that miRNA dysregulation evolves together with cognitive decline [134]. Nevertheless, peripheral miRNA alterations should not be assumed to directly reflect molecular changes occurring within the brain.

Beyond AD, miRNA dysregulation has also been associated with other neurodegenerative disorders. In ALS, miR-181 has emerged as a circulating miRNA associated with disease progression and mortality, while miRNA alterations in motor neurons have also been reported [135]. In relapsing-remitting MS, increased miR-106a-5p levels have been detected in cerebrospinal fluid and associated with oligoclonal bands, linking miRNA dysregulation with inflammatory processes occurring during early disease stages [136]. Distinct miRNA expression patterns have likewise been reported in PD, HD, and vascular-related cognitive disorders, further supporting the involvement of miRNA regulatory networks across neurodegenerative conditions [126,127,137].

### 4.7. Rare Neurological and Neurodevelopmental Diseases

Rare genetic disorders provide particularly informative examples of how disruption of miRNA biogenesis and function can affect nervous system development. Pathogenic variants affecting genes encoding components of the miRNA processing machinery, including *DROSHA*, *DGCR8*, *DICER1*, and genes encoding AGO proteins, have been associated with human disease, with variants in several of these genes linked to neurodevelopmental phenotypes. In particular, alterations affecting miRNA-processing proteins have been associated with intellectual disability, epilepsy, microcephaly, and other neurological manifestations, illustrating how disruption of global miRNA regulation can interfere with normal neural development [138].

miRNA dysfunction can also contribute to genetically defined neurodevelopmental disorders without directly affecting the miRNA processing machinery. In Fragile X syndrome, the fragile X messenger ribonucleoprotein (FMRP) interacts with miRNA-mediated regulatory mechanisms, including miR-125b and miR-132, to modulate dendritic spine morphology, synaptic plasticity, and expression of the NMDA receptor subunit NR2A. Similarly, miRNA-dependent regulatory mechanisms have been implicated in Rett syndrome, where interactions between miRNAs and MECP2-related signalling contribute to neuronal maturation and synaptic regulation [30,108]. Together, these disorders illustrate how abnormalities affecting either the miRNA machinery itself or its interaction with disease-associated genes can disrupt neuronal development and synaptic function.

### 4.8. Metabolic and Endocrine Disorders with Neurological Involvement

miRNAs may provide a molecular link between systemic metabolic dysregulation and neurological dysfunction. Numerous miRNAs regulate carbohydrate and lipid metabolism, insulin signalling, and energy homeostasis, and their dysregulation has been associated with metabolic disorders including type 2 diabetes, obesity, insulin resistance, and dyslipidaemia [139,140]. Some of these regulatory mechanisms also operate in the nervous system, providing potential links between metabolic dysfunction and neurodegenerative processes.

A particularly relevant example is miR-7. Experimental studies have shown that miR-7 directly targets the insulin receptor (INSR), insulin receptor substrate 2 (IRS-2), and insulin-degrading enzyme (IDE), thereby modulating insulin signalling and amyloid-β metabolism in the CNS. miR-7 expression was altered in the brain of diet-induced obese mice and increased in the brain of patients with Alzheimer’s disease, while its overexpression increased extracellular amyloid-β levels in neuronal cells and impaired its clearance by microglial cells [141]. The miR-34 family may provide an additional link between metabolic and neurodegenerative alterations, since its dysregulation has been associated with diabetes and obesity in peripheral metabolic tissues and with pathways regulating neuronal survival, synaptic plasticity, mitochondrial function, energy metabolism, and amyloid-β clearance in the brain [142].

miRNAs may also participate in interactions between endocrine and nervous systems. miRNA dysregulation has been described in multiple endocrine disorders, including diabetes mellitus, thyroid diseases, pituitary tumors, Cushing’s syndrome, and adrenal insufficiency [143]. Experimental evidence further suggests that extracellular miRNAs may contribute to endocrine–neural communication. In human adrenocortical H295R cells, exposure to the endocrine-disrupting compound DCOIT altered intracellular and extracellular miRNA profiles. Exosomes released by exposed cells subsequently modified neurite growth, neuronal differentiation, and gene expression in PC12 cells, providing experimental evidence that extracellular miRNAs can transmit signals from endocrine cells to neuronal cells [144]. These findings suggest that miRNAs may contribute to the molecular crosstalk linking metabolic and endocrine disturbances with alterations in nervous system function.

### 4.9. miRNAs as Diagnostic and Prognostic Biomarkers

Circulating miRNAs have emerged as potential biomarkers for neurological and neuropsychiatric disorders because they can be detected in accessible biological samples, including blood, plasma, serum, saliva, and cerebrospinal fluid. Individual miRNAs or combined signatures have been associated with diagnosis, disease progression, prognosis, and phenotypic stratification. Extracellular vesicles represent an additional source of candidate biomarkers, as their miRNA cargo may reflect pathological changes in their cells of origin [145].

Several studies illustrate their potential diagnostic value. In autism spectrum disorder, serum miR-197-5p and miR-500a-5p showed substantial diagnostic discrimination when evaluated as a combined signature [123], while plasma profiling in young children identified a four-miRNA signature with high discriminatory performance [124]. However, a larger multicenter study using salivary miRNAs showed more moderate diagnostic performance, emphasizing the importance of independent validation and clinically relevant comparison groups [121].

miRNAs may also provide prognostic information. In amyotrophic lateral sclerosis, circulating miR-181 has been associated with mortality risk and disease progression [135]. Altered circulating miRNA profiles have also been reported in epilepsy, although their clinical utility remains to be established [125].

From an analytical perspective, miRNA biomarker studies use different platforms depending on whether the objective is broad profiling or targeted quantification. High-throughput approaches, including next-generation sequencing (NGS) and microarrays, are commonly used during the discovery phase to identify candidate miRNAs and multi-miRNA signatures, whereas reverse transcription quantitative PCR (RT-qPCR) is widely used for targeted quantification and validation of selected candidates. Digital PCR (dPCR), including droplet digital PCR (ddPCR), provides an alternative approach based on sample partitioning and enables absolute quantification without the need for standard curves. This may be advantageous for low-abundance circulating miRNAs, although dPCR does not eliminate variability introduced during RNA extraction and reverse transcription [146]. Therefore, differences in analytical platform, extraction procedures, reverse-transcription protocols, amplification efficiency, and normalization strategies should be considered when comparing biomarker studies.

Pre-analytical variability represents an additional challenge for circulating miRNA biomarkers. miRNA profiles may differ substantially according to the biological matrix examined, and measurements obtained from whole blood, serum, plasma, saliva, cerebrospinal fluid, or extracellular-vesicle preparations should not be considered directly interchangeable. Sample collection and processing, centrifugation conditions, time to processing, storage conditions, freeze–thaw cycles, and RNA isolation procedures may all influence miRNA recovery. In blood-derived samples, haemolysis is particularly relevant because the release of intracellular miRNAs from blood cells may alter apparent circulating miRNA concentrations [147]. Standardized sample handling, assessment of sample quality, and appropriate normalization strategies are therefore essential to improve reproducibility across studies.

Importantly, promising diagnostic or prognostic results from initial studies require further analytical and clinical validation. Analytical validation requires demonstration that an assay measures the target miRNA with adequate sensitivity, specificity, precision, and reproducibility, whereas clinical validation requires confirmation that the biomarker reliably identifies or stratifies the intended clinical population. Validation should also account for potential biological and clinical sources of variability, including age, disease stage, treatment, comorbidities, and sample type. The differences in diagnostic performance observed among ASD studies using serum, plasma, and saliva illustrate the importance of these considerations [121,123,124].

Representative examples of miRNA candidates and signatures investigated for diagnostic or prognostic applications, together with their biological sample and analytical approach, are summarized in Table 13.

Despite these promising findings, substantial methodological and biological heterogeneity still limits direct comparison among studies and their translation into clinical practice. In particular, peripheral miRNA alterations should not be assumed to directly reflect molecular changes within the central nervous system. Clinical implementation will therefore require standardized pre-analytical and analytical workflows, robust normalization procedures, and independent validation in sufficiently large and clinically well-characterized cohorts.

### 4.10. Therapeutic Potential of miRNA Modulation

The ability of miRNAs to simultaneously regulate multiple components of disease-related molecular pathways makes them attractive therapeutic targets in neurological and neuropsychiatric disorders. Two major strategies are currently being investigated: inhibition of overexpressed miRNAs using antisense oligonucleotides or antagomirs, and replacement of deficient miRNAs using synthetic miRNA mimics. Both approaches have shown therapeutic potential in experimental models of CNS disorders [148,149].

Preclinical studies provide evidence that modulation of specific miRNAs can influence disease-related phenotypes. In experimental models of cognitive impairment and neurodegeneration, increasing miRNAs such as miR-132 and decreasing miR-34a and miR-124 have been associated with improved cognitive or synaptic outcomes [150]. Similarly, miRNA modulation is being explored in neuropsychiatric disorders, including major depressive disorder and autism spectrum disorder, where dysregulated miRNAs regulate pathways involved in neuroplasticity, neuroinflammation, neuronal development, and synaptic function [115,151,152,153]. However, much of this evidence remains mechanistic or preclinical rather than demonstrating therapeutic efficacy in humans. To provide an integrative translational overview connecting physiological developmental pathways with pathological alterations and experimental therapeutic outcomes, Table 14 summarizes the key microRNAs discussed across this review.

The translation of these approaches to CNS disorders remains challenging. Because individual miRNAs can regulate numerous transcripts, therapeutic modulation may produce unintended off-target effects. Efficient and cell-specific delivery across the blood–brain barrier represents an additional major limitation. Viral vectors, lipid and polymeric nanoparticles, and exosome-based delivery systems are therefore being investigated to improve stability, CNS penetration, and cellular targeting [148,149]. Despite substantial preclinical progress, clinical evaluation of miRNA-based therapies for CNS disorders remains limited, and further studies are required to establish their long-term efficacy and safety.

**Table 14:** Key microRNAs in neural development and neurological disorders: physiological roles, pathology, models, and therapeutics.

miRNA	Physiological Neurodevelopmental Role	Experimental Model and Pathological Mechanism	Associated Disorders and Pathophysiological Impact	References
miR-9	Neurogenic switch; brain patterning; neuronal migration and morphogenesis	Models: Human post-mortem brain, CSF, and leukocytes; HD (R6/2), AD (APP/PS1), and ALS (SOD1) rodent models; SCZ iPSC-derived NPCs. Mechanisms: Nuclear REST derepression in HD; Aβ neurotoxicity and amyloidogenesis in AD; NPC migratory arrest in SCZ; motor neuron loss in ALS. Rescued via mimics/antagomirs.	HD: ↓ Reduced in cortex, striatum, and blood; derepresses REST/CoREST, causing transcriptional silencing of neuronal genes.	[30,127,128]
			AD: ↓ Decreased in cortex and CSF; derepresses BACE1, impairs autophagy, and exacerbates Aβ-driven neurotoxicity.	[127,128,129]
			SCZ: ↓ Reduced in DLPFC and NPCs; disrupts migration, cell adhesion, and DRD2 networks.	[39]
			ALS: ↑ Elevated in spinal motor neurons; destabilizes neurofilament mRNAs and impairs neurogenesis.	[126,127]
miR-34	Cell cycle exit and differentiation; neuronal maturation and morphogenesis; cortical architecture and ciliogenesis	Models: Human brain (cortex, hippocampus, substantia nigra); AD (APP/PS1, SAMP8) and HD (R6/2) rodents; patient iPSC-NPCs. Mechanisms: p53 activation, vesicle release failure (SYT1, VAMP2), blunted AMPA/NMDA currents, and SIRT1 depletion. Antagomirs (anti-miR-34a/c) restore LTP, spines, and cognition.	AD: ↑ miR-34a/c brain; targets SIRT1, VAMP2, SYT1, and AMPAR/NMDAR, driving spine loss, LTP blunting, tau hyperphosphorylation, and amyloidogenesis.	[127,129,142,150]
			PD: ↓ miR-34b/c; ↑ miR-34a: Nigral miR-34b/c loss elevates SNCA and mitochondrial stress; astrocytic miR-34a represses Bcl-2, promoting dopaminergic apoptosis.	[30,126,127,128]
			HD: ↓ miR-34a: Striatal downregulation alters the p53/SIRT1 neuroprotective network.	[30,126,127,128]
			BD, SCZ: ↑ miR-34a: Elevated in cortex and blood; targets *ANK3*, *CACNB3*, and *SYT1*, impairing NPC differentiation and synaptic maturation.	[50,151]
miR-107	Neurogenic timing, progenitor boundary specification, survival, and neuritogenesis	Models: Human brain (cortex, hippocampus) and AD/MCI/SCZ biofluids; zebrafish embryos; hESC-derived neurons. Mechanisms: Early BACE1 derepression accelerating Aβ deposition; Dicer–miRNA checkpoint breakdown; loss of BDNF and FGF7/Akt signaling. Mimics reduce Aβ and neurotoxicity.	AD: ↓ cortex/hippocampues/CSF/plasma: derepresses BACE1 (↑ Aβ deposition) and alters *CDK5R1* (p35), promoting CDK5-mediated tau pathology.	[127,128,130,149]
			SCZ: ↑/altered serum: Dysregulated in DLPFC and peripheral blood; impacts cortical neuronal migration and synaptic connectivity genes.	[107,109,110]
			ASD: ↓/altered: Imbalanced in brain and blood; targets *CAV1*, impairing lipid homeostasis and neural insulin signaling.	[113,117,139,140]
miR-124	Neuronal fate consolidation; alternative splicing switch; gliogenesis repression; morphogenesis and connectivity	Models: Human brain (cortex, substantia nigra, spinal cord) and biofluids; AD (APP/PS1), PD (MPTP), HD (R6/2), and MS (EAE) rodents; human iPSC-derived neurons. Mechanisms: Defective BACE1/tau clearance and AMPAR trafficking; microglial hyperactivation (MEKK3/p38/C/EBPα); blunted neurogenesis. Agomirs, mimics, or nanoparticles rescue LTP and behavioral deficits.	AD: ↓ cortex/hippocampus/CSF: Relieves *BACE1* repression (↑ Aβ deposition), alters *PTPN1* (AMPAR endocytosis), and ↑ tau phosphorylation via CAV1/GSK-3β.	[126,127,128,149,150]
			PD: ↓ plasma: Disinhibits Bim (apoptosis) and MEKK3/p62/p38 cascades, provoking microglial activation and dopaminergic loss.	[27,30,126,127,149]
			FTD, ALS: ↓ cortex/spinal cord: Represses GluR2 (driving excitotoxicity and social deficits in FTD); exosomal secretion marks ALS motor severity.	[126,127,150]
			HD: ↓ striatum: Blunts compensatory adult neurogenesis.	[27,56,126,127,149]
			MS: ↓ microglia: Promotes pro-inflammatory M1 phenotype via C/EBP-α and PU.1 derepression.	[27,56,126,127,149]
miR-132	Activity-dependent synaptogenesis, axon outgrowth and guidance, cortical integration and migration, subtype specification	Models: Human brain (temporal cortex, DLPFC, hippocampus) and biofluids; miR-132/212 KO, AD (APP/PS1, 3xTg), and Tau (P301S) mice; seizure models; patient iPSC-neurons. Mechanisms: miR-132 loss triggers tau aggregation, spine collapse, and memory deficits. Lentiviral delivery or mimics restore synaptic transmission, attenuate tauopathy, and rescue cognition.	AD, Tauopathies: ↓ brain/CSF: Derepression of *MAPT*, *ITPKB*, and *GSK3β* drives tau hyperphosphorylation, Aβ burden, and PTEN/FOXO3 apoptosis.	[126,127,131,132]
			SCZ: ↓ cortex/serum: Blunts NMDAR depolarization and alters neurodevelopmental synaptic networks.	[26,106,107,110]
			TLE: ↑ hippocampus: Post-seizure induction hyperactivates BDNF–TrkB–CREB signaling, driving aberrant spine sprouting and network hyperexcitability.	[80,109,125]
			ASD, Rett Syndrome: altered neocortex: Disrupts the homeostatic CREB–miR-132–MeCP2 axis, impairing synaptic pruning and excitatory/inhibitory balance.	[26,30,108,116,117,118]
miR-134	Progenitor proliferation/radial migration, activity-dependent dendritogenesis, spine restraint, and homeostatic depression	Models: Human surgical brain tissue (hippocampus/cortex) and biofluids; seizure rodents (kainate, pilocarpine, PPS); Aβ-treated hippocampal slices. Mechanisms: MEF2-driven miR-134 elevation lowers seizure thresholds; Ant-134 antagomir suppresses seizures (>90%), restoring spine structure, LTP, and memory.	TLE, SE: ↑: LIMK1/PUM2/CREB repression driving aberrant spine remodeling and network hyperexcitability.	[28,80,109,125]
			AD: ↑ cortex/hippocampus: Represses LIMK1 and CREB, impairing synaptic tagging, LTP, and memory; inversely regulated by neuroprotective SIRT1.	[133,150]
			BD, MDD, SCZ: altered/↓ brain/blood: Dysregulated in DLPFC and blood; plasma levels track mania treatment response and correlate with depressive severity.	[107,108,110,151]
miR-137	Neural stem cell fate and differentiation, epigenetic chromatin orchestration, dendritic morphogenesis and synaptogenesis	Models: Human brain (DLPFC, hippocampus); 1p21.3 microdeletion cohorts; *Mir137* het/cKO mice; patient iPSC-neurons; ketamine model. Mechanisms: Active-zone vesicle depletion, presynaptic LTP loss, blunted Nrg1α/BDNF–Akt–mTOR signaling, and striatal *Pde10a* upregulation. Rescued by PDE10A inhibitor (MP-10) or miRNA restoration.	SCZ: ↓: GWAS locus (rs1625579) reduces DLPFC miR-137, targeting *TCF4*/*CACNA1C*; disrupts vesicle release (SYT1/CPLX1), Nrg1–ErbB4, and mossy fiber LTP.	[3,26,27,28,109,110]
			ASD, 1p21.3 Microdeletion Syndrome: ↓: Microdeletion causes ID and ASD; derepresses *Pde10a* and *Shank2*, driving repetitive behaviors deficits.	[3,26,27,28,112,117]
			BD: altered: Linked to *MIR137HG* enhancer variants, disrupting fronto-striatal and fronto-amygdala functional connectivity.	[28,106,112,151]
			AD: ↓ cortex/hippocampus: Relieves repression of *CACNA1C*, exacerbating aberrant calcium influx and Aβ-induced tau hyperphosphorylation.	[30,150]
miR-181	Structural neuronal maturation, dendritic arborization, cerebellar neurogenesis and survival	Models: Human plasma cohorts (ALS); human brain (temporal cortex, DLPFC) and blood (SCZ); APP/PS1 mice (AD); status epilepticus rodents (TLE). Mechanisms: Axonal biomarker shedding in ALS; apoptotic and synaptic pathway dysregulation in SCZ; CRMP2 microtubule breakdown and mitochondrial ROS in AD; GluA2 internalization in TLE. Antagomirs attenuate seizure-induced death.	ALS: ↑ plasma: Predicts >2-fold higher mortality risk and progression as an independent biomarker complementary to neurofilament light chain.	[149]
			SCZ: ↑ cortex/blood: Targets *BCL2*, *AKT1*, and *PTEN*, altering apoptosis and synaptic transmission; normalizes post-antipsychotic response.	[3,107,109,110,151]
			AD: ↓ cortex/hippocampus: Targets CRMP2 and mitochondrial *MT-CO1*, exacerbating tau hyperphosphorylation, neurite retraction, and respiratory chain failure.	[30,131,133,140]
			TLE: ↑ foci/post-SE: Represses GluA2 (AMPAR) and GABAAα1, driving synaptic hyperexcitability and seizure-induced cell death.	[28,125]
miR-185	Neural stem cell fate balance, gliogenesis regulator, dendritic and spine morphogenesis	Models: Human brain/blood (22q11DS/SCZ); patient iPSC-neurons; Df(16)A^+^/^−^ mice. Mechanisms: DGCR8 microprocessor synergism; Golgi trafficking defects via Mirta22/Emc10; RhoA/Cdc42 imbalance. Genetic replenishment rescues dendritic and spine deficits.	DiGeorge Syndrome, SCZ: ↓: 22q11.2 loss and SCZ deplete miR-185, derepressing Mirta22/Emc10, RhoA, and Cdc42 to cause spine loss and connectivity deficits.	[3,14,23,93,110,151]
			ASD: ↓: Depleted in patient cortical neurons and pediatric blood/PBMCs; impairs synaptogenesis and calcium signaling.	[107,108,115]
			MDD, Suicide Risk: ↑ frontopolar cortex: Directly targets astrocytic TrkB-T1, blunting BDNF-evoked Ca^2+^ transients and neuroprotection.	[151]
miR-191	Neural differentiation, activity-dependent synaptic plasticity, neuritogenesis and outgrowth	Models: Human brain (posterior cingulate cortex) and serum (AD, PD, MS); PSD rat model; SH-SY5Y and primary hippocampal neurons. Mechanisms: DAPK1 derepression accelerating caspase activation and tauopathy in AD; BDNF suppression impairing plasticity and survival in PSD and MS. Mimics rescue neurite outgrowth and attenuate Aβ toxicity.	AD, Tauopathies: ↓ cortex: Inversely correlates with neurofibrillary tangle burden; ↓ DAPK1, ↑ tau hyperphosphorylation, Aβ generation, and apoptosis.	[96,129,131,150]
			MS: ↑ serum: Elevated in RRMS and PPMS; correlates with EDSS disability, repressing BDNF and facilitating immune/neuronal apoptosis.	[127]
			PD: ↑ serum: Significantly elevated in patient serum, acting as a circulating diagnostic discriminator.	[137]
			Cognitive Impairment: ↑ hippocampal: Upregulated following paradoxical sleep loss; directly represses BDNF, causing spatial learning and memory deficits.	[150]
miR-200	Progenitor cell-cycle exit, suppression of neural stemness, late maturation and olfactory neurogenesis	Models: Human brain (PCC) and biofluids/PBMCs (AD, SCZ, ASD); mouse neurogenesis in vivo; PC12 and primary neurons. Mechanisms: BACE1 repression and Aβ synaptotoxicity (AD); Zeb2-mediated survival failure; defective progenitor commitment and synaptic maintenance (SCZ/ASD).	AD, Resilience: altered: miR-200b/c targets *APP*/*BACE1* to blunt Aβ toxicity; cortical miR-429 upregulation marks cognitive resilience despite AD pathology.	[129,131,149,150]
			SCZ, MDD: ↓: Reduced in PBMCs of SCZ cohorts; altered in depression, impacting Wnt/Notch neurodevelopmental and cell-adhesion networks,	[3,23,107,110,151]
			ASD: altered circulating: Aberrant salivary and serum miR-141-3p in pediatric cohorts; linked to CNVs disrupting neural connectivity genes.	[107,112,116,117]
miR-218	Motor neuron specification, cortical migration/laminar positioning, and circuit maturation	Human sALS/fALS (cord/cortex); LCM interneurons; Mir218 dKO mice; stress models. Mechanisms: Fatal ALS-like neuromuscular failure; Kv10.1 alters MN firing; DCC impairs axon guidance. Rescued by miR-218 restoration or Kv10.1 blockade.	ALS: ↓: Motor neurons depletion; MIR218 loss derepresses Kv10.1 (*KCNH1*), driving MN hyperexcitability, NMJ dissolution, and progressive paralysis.	[126,127,135,149]
			MDD: ↑ mPFC: Stress-induced elevation in prefrontal cortex; directly targets Netrin-1 receptor DCC, disrupting mesocortical synaptic connectivity.	[151,153]
			SCZ: ↓: Downregulated in cortical parvalbumin interneurons (STG), impairing inhibitory microcircuit pacing and auditory sensory gating.	[125,151]
miR-344	Embryonic neural restriction, nuclear localization in immature neurons	Rodent embryonic/postnatal brain; ESCs and immature primary neurons. Spatiotemporally restricted to early neurodevelopment; disease mechanisms uncharacterized.	No established disease association in human neurological or neuropsychiatric disorders to date. Dysregulation patterns and pathological consequences have not been formally identified in clinical neuropathology or standard animal disease models.	[104,105]

22q11DS, 22q11.2 deletion syndrome; 3xTg, triple-transgenic mouse model; AD, Alzheimer’s disease; ALS, amyotrophic lateral sclerosis; AMPA, α-amino-3-hydroxy-5-methyl-4-isoxazolepropionic acid; AMPAR, AMPA receptor; APP/PS1, amyloid precursor protein/presenilin 1 transgenic mouse model; ASD, autism spectrum disorder; BD, bipolar disorder; BDNF, brain-derived neurotrophic factor; cKO, conditional knockout; CNVs, copy number variants; CREB, cAMP response element-binding protein; CSF, cerebrospinal fluid; Df(16)A, mouse model carrying a chromosomal deletion syntenic to the human 22q11.2 deletion; DLPFC, dorsolateral prefrontal cortex; dKO, double knockout; EAE, experimental autoimmune encephalomyelitis; EDSS, Expanded Disability Status Scale; ESCs, embryonic stem cells; fALS, familial amyotrophic lateral sclerosis; FTD, frontotemporal dementia; GABAA, γ-aminobutyric acid type A receptor; GWAS, genome-wide association study; HD, Huntington’s disease; hESC, human embryonic stem cell; ID, intellectual disability; iPSC, induced pluripotent stem cell; KO, knockout; LCM, laser capture microdissection; LTP, long-term potentiation; MCI, mild cognitive impairment; MDD, major depressive disorder; MN, motor neuron; MP-10, selective phosphodiesterase 10A inhibitor; mPFC, medial prefrontal cortex; MPTP, 1-methyl-4-phenyl-1,2,3,6-tetrahydropyridine; MS, multiple sclerosis; NMDA, N-methyl-D-aspartate; NMDAR, NMDA receptor; NMJ, neuromuscular junction; NPCs, neural progenitor cells; P301S, tau proline-to-serine substitution at residue 301; PBMCs, peripheral blood mononuclear cells; PC12, rat pheochromocytoma-derived cell line; PCC, posterior cingulate cortex; PD, Parkinson’s disease; PPMS, primary progressive multiple sclerosis; PPS, perforant pathway stimulation; PSD, paradoxical sleep deprivation; R6/2, transgenic mouse model of Huntington’s disease; REST, RE1-silencing transcription factor; ROS, reactive oxygen species; RRMS, relapsing-remitting multiple sclerosis; SAMP8, senescence-accelerated mouse prone 8; sALS, sporadic amyotrophic lateral sclerosis; SCZ, schizophrenia; SE, status epilepticus; SH-SY5Y, human neuroblastoma-derived cell line; STG, superior temporal gyrus; TLE, temporal lobe epilepsy; TrkB, tropomyosin receptor kinase B.

## 5. Conclusions and Future Perspectives

miRNAs constitute an important regulatory layer in nervous system development, acting across multiple and partially overlapping stages ranging from neural progenitor proliferation and differentiation to neuronal migration, maturation, synaptogenesis, and activity-dependent plasticity. The evidence reviewed here also illustrates the diversity of their functions: while miRNAs such as miR-9, miR-124, miR-132, and miR-137 are supported by substantial mechanistic evidence, the roles of other developmentally regulated miRNAs remain less well characterized. Importantly, miRNA function is highly dependent on developmental stage, cell type, brain region, and expression level, emphasizing that their biological effects cannot be understood independently of their cellular and developmental context.

The widespread dysregulation of miRNAs across neurodevelopmental, neuropsychiatric, and neurodegenerative disorders further supports their relevance to nervous system disease. Nevertheless, altered miRNA expression should not necessarily be interpreted as evidence of a causal role, since these changes may also represent secondary or compensatory responses to pathological processes. Similarly, circulating miRNA profiles provide promising opportunities for minimally invasive biomarkers but do not necessarily reflect molecular alterations within the CNS. Future studies should therefore integrate mechanistic evidence from experimental models with longitudinal analyses of well-characterized human cohorts and, where possible, parallel assessment of central and peripheral biological samples.

From a translational perspective, miRNAs offer complementary opportunities as biomarkers and therapeutic targets. However, clinical implementation will require improved methodological standardization, independent and multicenter validation of biomarker signatures, and demonstration of their added value over established clinical markers. For miRNA-based therapeutics, major priorities include achieving cell- and tissue-specific delivery, improving blood–brain barrier penetration, minimizing off-target effects, and establishing long-term safety. Advances in single-cell and spatial approaches, together with improved experimental models and delivery technologies, should help define the precise spatiotemporal functions of individual miRNAs and determine which regulatory networks represent clinically actionable targets. These developments will be essential for translating the expanding knowledge of miRNA biology into diagnostic and therapeutic applications in neurological and neuropsychiatric disorders.

## Figures and Tables

**Table 1:** Main molecular targets and functions of miR-9.

Target/Pathway	Main Function	Role of miR-9 Regulation	Mechanistic Evidence	References
FoxG1	Cortical development and early-born neuronal populations	Restricts FoxG1 expression during telencephalic neurogenesis	Direct 3′UTR targeting; reporter assays and miR-9 loss-of-function in mice; stage-dependent regulation	Shibata et al., 2011 [35]
Hes1/Her6/Her9	Neural progenitor maintenance	Controls progenitor maintenance versus differentiation	Experimental perturbation and expression-dynamics analysis; mathematical modelling; target-specific evidence varies	Tan et al., 2012 [36]; Soto et al., 2022 [31]
TLX/Nr2e1	Neural stem-cell self-renewal	Promotes transition towards neuronal differentiation	3′UTR-mediated, context-dependent regulation; reporter assays and mouse loss-of-function studies	Davila et al., 2014 [33]; Shibata et al., 2011 [35]
Gsh2	Proliferation and differentiation in the subpallium	Regulates progenitor proliferation/differentiation balance	Direct repression; wild-type/mutant 3′UTR reporter assays and in vivo protein analysis	Shibata et al., 2011 [35]
Diap1/Map1b	Cytoskeletal organization and neurite development	Contributes to neuronal morphogenesis	Direct repression; 3′UTR reporter, protein expression and functional rescue assays	Lin et al., 2017 [37]
REST	Repression of neuronal genes	miR-9 repression of REST reinforces differentiation	Direct REST targeting reported; reciprocal transcriptional repression and cell-based validation	Laneve et al., 2010 [34]
HDAC4/Mef2C	Neuronal differentiation	Sustains Mef2C activity and reinforces miR-9 expression	HDAC4 repression and MEF2C-dependent transcriptional feedback; knockdown, promoter-reporter and ChIP assays	Davila et al., 2014 [33]

**Table 2:** Main molecular targets and functions of miR-34.

Target/Pathway	Main Function	Role of miR-34 Regulation	Mechanistic Evidence	References
SIRT1	Neuronal differentiation and neurite extension	Promotes neuronal differentiation and neurite development	SIRT1 protein downregulation and siRNA-mediated functional validation; SIRT1-dependent neurite outgrowth but SIRT1-independent neuronal differentiation	Aranha et al., 2011 [47]
Syt1/Stx1A	Synaptic vesicle release	Modulates synaptic maturation and plasticity	Direct Syt1 targeting (3′UTR luciferase assay) and partial functional rescue; Stx1A expression regulation reported	Agostini et al., 2011 [46]; Morgado et al., 2015 [43]
Atg9a	Autophagic flux	Regulates autophagy during neuronal development	Direct targeting (3′UTR luciferase assay); protein downregulation, altered autophagy markers (LC3-II/p62), and partial pharmacological rescue	Morgado et al., 2015 [43]
JAM-A	Cell adhesion and radial-glial division	Contributes to cortical architecture	Direct JAM-A targeting by miR-449a (wild-type/mutant binding-site assays); miR-34/449 family knockout and functional rescue experiments	Fededa et al., 2016 [48]
DCX	Neuroblast migration and dendritic development	Influences migration and morphological maturation	Direct targeting (wild-type/mutant 3′UTR luciferase assays); mRNA/protein downregulation and in vivo neuroblast migration and dendritic morphology analyses	Mollinari et al., 2015 [42]
p53 pathway	Stress response and apoptosis	Feedback regulation of p53-dependent responses	p53-dependent miR-34a induction and miR-34a-mediated p53 downregulation in PC12 cells; functional feedback demonstrated, but direct targeting not validated	Jauhari et al., 2018 [44]
*ANK3*/*CACNB3*	Neuronal and synaptic function	Altered regulation affects synaptogenesis	Direct targeting (3′UTR luciferase assays); inverse expression correlations and anti-miR-34a-mediated target upregulation; neuronal differentiation and morphology assays	Bavamian et al., 2015 [50]

**Table 3:** Main molecular targets and functions of miR-107.

Target/Pathway	Main Function	Role of miR-107 Regulation	Mechanistic Evidence	References
Dicer	miRNA processing	Regulates Dicer levels and the biogenesis of specific neural miRNAs	Direct Dicer targeting validated by in vivo reporter assays; miR-107 loss-of-function and analysis of downstream miRNA biogenesis in zebrafish	Ristori et al., 2015 [51]
miR-9 biogenesis	Progenitor maintenance/differentiation	Dicer regulation influences mature miR-9 levels	Indirect regulation via Dicer; miR-107 loss-of-function increases Dicer stability and mature miR-9 accumulation, with associated neurogenic changes in zebrafish	Ristori et al., 2015 [51]
BDNF	Survival and neurite development	Excess miR-107 may reduce BDNF under neurotoxic conditions	Direct targeting (wild-type/mutant 3′UTR luciferase assays); inverse BDNF expression and siRNA-mediated functional validation in ketamine-exposed neurons	Jiang et al., 2019 [53]

**Table 4:** Main molecular targets and functions of miR-124.

Target/Pathway	Main Function	Role of miR-124 Regulation	Mechanistic Evidence	References
REST/SCP1	Repression of neuronal genes	Reinforces neuronal gene-expression programme	Direct SCP1 targeting (3′UTR luciferase assay); indirect modulation of REST-associated transcriptional repression	Åkerblom, 2014 [58]; Visvanathan et al., 2007 [62]
PTBP1/PTBP2	Alternative RNA splicing	Promotes neuron-specific splicing	Direct 3′UTR targeting of PTBP1 and PTBP2 (luciferase assays and site-directed mutagenesis); PTBP1 knockdown and re-expression experiments establish its role in neuron-specific splicing and differentiation. PTBP2 is indirectly upregulated through PTBP1-dependent splicing and NMD	Åkerblom, 2014 [58]; Makeyev et al., 2007 [63]
SOX9	Glial specification	Promotes neuronal differentiation through SOX9 repression	Direct SOX9 targeting (3′UTR reporter assays); SOX9 gain- and loss-of-function experiments demonstrating its role in neuronal versus glial differentiation in the adult mouse SVZ	L. C. Chen et al., 2009 [64]
NeuroD1	Early neurogenesis	Represses neuronal differentiation and promotes progenitor proliferation during early optic vesicle development	Direct NeuroD1 targeting (wild-type/mutant 3′UTR luciferase assays); gain- and loss-of-function experiments and NeuroD1-mediated functional rescue in Xenopus embryos	K. Liu et al., 2011 [65]
ROCK1/RhoG/Cdc42 RhoG–ELMO/ Dock180/Rac1–Cdc42	Cytoskeletal organization	Promotes axonal and dendritic branching	Direct RhoG targeting (3′UTR EGFP reporter assays, seed-site mutagenesis and endogenous protein regulation); gain-/loss-of-function and rescue experiments establish RhoG-dependent control of neuronal branching via Rac1 and Cdc42 signalling	Franke, 2012 [66]

**Table 5:** Main molecular targets and functions of miR-132.

Target/Pathway	Main Function	Role of miR-132 Regulation	Mechanistic Evidence	References
*Rasa1*	Axonal development	Facilitates axon extension	Direct *Rasa1* targeting (3′UTR luciferase assay and target-site protection); miR-132 gain-/loss-of-function, *Rasa1* knockdown and 3’UTR-independent overexpression demonstrate *Rasa1*-dependent regulation of axon extension	Hancock et al., 2014 [74]
MeCP2/BDNF	Neural maturation and homeostasis differentiation and maturation	Represses MeCP2 expression and is induced by BDNF	miR-132-mediated MeCP2 downregulation and partial phenotypic replication by MeCP2 knockdown; BDNF-induced miR-132 expression	D. Chen et al., 2018 [72]; Yang et al., 2012 [75]
Nurr1	Dopaminergic differentiation	Suppresses dopaminergic differentiation through Nurr1 downregulation	Direct Nurr1 targeting (3′UTR luciferase assays and site-directed mutagenesis); miR-132 gain-/loss-of-function experiments demonstrate inhibition of dopaminergic differentiation	D. Chen et al., 2018 [72]; Yang et al., 2012 [75]
Foxp2	Cortical neuronal migration	Contributes to correct radial migration	Direct Foxp2 3′UTR targeting (in vivo luciferase assays and site-directed mutagenesis); miRNA-resistant Foxp2 overexpression impairs cortical neuronal migration	D. Chen et al., 2018 [72]; Clovis et al., 2012 [76]

**Table 6:** Main molecular targets and functions of miR-134.

Target/Pathway	Main Function	Role of miR-134 Regulation	Mechanistic Evidence	References
Pum2	Translational regulation	Regulates activity-dependent dendritogenesis	miR-134 overexpression reduces Pum2 protein levels (Western blot) and impairs dendritic growth in vivo	Christensen, 2010 [83]
Nanog/LRH1	Stem-cell self-renewal	Facilitates transition towards differentiation	Direct 3′UTR targeting (luciferase assays), reduced Nanog/LRH1 protein expression, and RNAi-mediated knockdown promoting embryonic stem-cell differentiation	Tay et al., 2008 [81]
Dcx	Neuronal migration	Restricts radial migration	Direct *Dcx* 3′UTR targeting (luciferase assays) and protein downregulation; in utero miR-134 overexpression impairs radial migration, rescued by Dcx re-expression	Gaughwin et al., 2011 [84]
Chrdl-1	BMP signalling	Influences progenitor survival and differentiation	Direct *Chrdl1* 3′UTR targeting (luciferase assays) and protein downregulation; RNAi, rescue and BMP-4 response assays support regulation of neural progenitor proliferation and survival	Gaughwin et al., 2011 [84]

**Table 7:** Main molecular targets and functions of miR-137.

Target/Pathway	Main Function	Role of miR-137 Regulation	Mechanistic Evidence	References
TLX/LSD1	Neural stem-cell proliferation and differentiation	Promotes the transition from proliferation to neuronal differentiation through the miR-137–TLX–LSD1 regulatory loop	Direct *Kdm1a* targeting (3′UTR luciferase assays and site-directed mutagenesis); TLX-mediated transcriptional repression of miR-137 (ChIP and reporter assays); functional rescue by LSD1 in vivo	Sun et al., 2011 [85]
EZH2	Adult hippocampal neurogenesis and epigenetic regulation	Regulates neural stem-cell proliferation and differentiation through repression of EZH2	Direct *Ezh2* targeting (3′UTR luciferase assays and target-site deletion); endogenous protein downregulation and functional rescue of neuronal differentiation by EZH2 re-expression in adult neural stem cells	Szulwach et al., 2010 [86]
Mib1	Neuronal maturation and dendritic development	Increased miR-137 activity impairs neuronal maturation and dendritic morphogenesis through Mib1 repression	Direct *Mib1* targeting (3′UTR luciferase assays and target-site mutagenesis); endogenous protein downregulation, Mib1 knockdown and partial rescue of dendritic defects by Mib1 re-expression in cultured neurons	Smrt et al., 2010 [87]

**Table 8:** Main molecular targets and functions of miR-181.

Target/Pathway	Main Function	Role of miR-181 Regulation	Mechanistic Evidence	References
α1ACT–miR-181 axis	Cerebellar neurodevelopment	α1ACT induces miR-181 expression	α1ACT-dependent miR-181 upregulation validated by miRNA profiling and TaqMan assays in transgenic mouse cerebellum; downstream pathway involvement inferred from integrated miRNA–mRNA analysis	Wei et al., 2022 [89]
IGF-1–CREB1	Dendritic growth and neuronal maturation	miR-181a represses CREB1 and limits dendritic growth	Direct *Creb1* mRNA targeting (3′UTR luciferase assays and site-directed mutagenesis); protein regulation, IGF-1-dependent miR-181a downregulation, and dendritic effects demonstrated by gain-/loss-of-function assays without CREB1-specific rescue	Liu et al., 2013 [90]

**Table 9:** Main molecular targets and functions of miR-185.

Target/Pathway	Main Function	Role of miR-185 Regulation	Mechanistic Evidence	References
Neural progenitor differentiation pathway	Neural progenitor proliferation and gliogenesis	Restricts progenitor proliferation and promotes glial differentiation	miR-185-5p gain-/loss-of-function assays demonstrate reduced neural progenitor proliferation and enhanced astroglial differentiation in vitro; antagomir experiments confirm these effects in vivo	Feng et al., 2024 [91]
Mirta22/Emc10	Dendritic morphogenesis	Repression of Mirta22/Emc10 regulates dendritic complexity	Direct *Emc10* mRNA targeting (3′UTR luciferase assays and site-directed mutagenesis); miR-185 gain-/loss-of-function and genetic rescue experiments demonstrate regulation of dendritic and spine morphology in a 22q11.2 deletion mouse model	Xu et al., 2013 [93]

**Table 10:** Main molecular targets and functions of miR-191.

Target/Pathway	Main Function	Role of miR-191 Regulation	Mechanistic Evidence	References
Neural differentiation-associated expression	Neural progenitor differentiation	Expression increases during neural differentiation	Expression profiling during neural differentiation of mouse P19 cells; association with neural progenitor differentiation, without direct target validation or functional evidence of causality	Huang et al., 2009 [94]
NMDA receptor-dependent activity	Neuronal activity and plasticity	Expression is modulated following NMDA receptor-dependent stimulation	Activity-dependent miR-191 upregulation demonstrated by LNA-based miRNA profiling following NMDA stimulation; receptor dependence supported by APV blockade	van Spronsen et al., 2013 [95]
DAPK1	Neurite development and neuronal function	Promotes neurite outgrowth through DAPK1-related mechanisms	miR-191-5p overexpression enhances neurite outgrowth in RA-differentiated SH-SY5Y cells; direct *DAPK1* mRNA targeting validated by wild-type/mutant 3′UTR luciferase assays and protein downregulation	Wang et al., 2022 [96]

**Table 11:** Main molecular targets and functions of miR-200 family.

Target/Pathway	Main Function	Role of miR-200 Family Regulation	Mechanistic Evidence	References
Sox2/E2F3	Neural progenitor cell-cycle exit and differentiation	Promotes cell-cycle exit and neuronal differentiation	Direct *Sox2*/*E2f3* mRNA targeting (wild-type/mutant 3′UTR luciferase assays); miR-200 gain-/loss-of-function supports neuronal differentiation, while promoter-reporter assays demonstrate SOX2/E2F3-dependent activation of miR-200c/141 transcription	Peng et al., 2012 [97]
SOX2/KLF4	Progenitor maintenance and neuronal differentiation	Reduces stemness-associated transcription factors and promotes differentiation	miR-200b overexpression reduces *Sox2*/*Klf4* mRNA and SOX2/KLF4 protein levels (RT-qPCR and Western blot); gain-/loss-of-function assays support neuronal differentiation	Pandey et al., 2015 [98]
Zeb2	Late neuronal differentiation and maturation	Repression promotes neuronal maturation	*Zeb2* 3′UTR luciferase repression by miR-200; in vivo gain-/loss-of-function, Zeb2 protein downregulation, Zeb2 re-expression rescue and conditional Zeb2 deletion support its role in postnatal neuronal maturation	Beclin et al., 2016 [99]

**Table 12:** Main molecular targets and functions of miR-218.

Target/Pathway	Main Function	Role of miR-218 Regulation	Mechanistic Evidence	References
Satb2	Cortical neuronal identity and morphogenesis	Regulates radial migration, morphogenesis, and cortical distribution	miR-218 overexpression reduces SATB2 expression and disrupts cortical neuronal migration and morphogenesis in vivo; SATB2 re-expression rescues these defects	Jiang et al., 2023 [100]
Interneuron and neural progenitor gene programmes	Motor neuron specification	Suppresses non-motor neuron programmes downstream of Isl1–Lhx3	Direct targeting of interneuron-associated genes (RISC-trap, wild-type/mutant 3′UTR luciferase assays and in vivo fluorescent reporters); miR-218 gain-/loss-of-function demonstrates suppression of interneuron differentiation and supports ISL1–LHX3-dependent motor-neuron specification	Thiebes et al., 2015 [101]
Kif21b/Syt13	Hippocampal circuit assembly	Regulates early neuronal activity and network maturation	*Kif21b*/*Syt13* derepression following miR-218 inhibition (RNA-seq and expression validation); combined target-site blockade partially reproduces the altered hippocampal network activity, supporting their functional involvement in early circuit assembly	Taylor et al., 2023 [102]
Synaptic gene networks	Dopaminergic neuronal function	Regulates neuronal excitability and dopamine release	miR-218 loss-of-function alters dopaminergic neuronal excitability and dopamine release (electrophysiology and amperometry); target prediction, pathway enrichment and RT-qPCR implicate synaptic gene networks	Pulcrano et al., 2023 [103]

**Table 13:** Representative miRNA candidates and signatures investigated as diagnostic or prognostic biomarkers in neurological and neurodevelopmental disorders.

miRNA Candidate/ Signature	Disorder	Biological Sample	Potential Clinical Application	Analytical Approach	References
miR-197-5p + miR-500a-5p	Autism spectrum disorder	Serum	Diagnostic discrimination	Stem-loop RT-qPCR	T. Kichukova et al., 2021 [123]
miR-4433b-5p, miR-15a-5p, miR-335-5p + miR-1180-3p	Autism spectrum disorder	Plasma	Early diagnostic classification	Small-RNA sequencing	S. Salloum-Asfar et al., 2025 [124]
Salivary multi-miRNA signature	Autism spectrum disorder	Saliva	Diagnostic classification	High-throughput small-RNA sequencing	S. D. Hicks et al., 2020 [121]
miR-181	Amyotrophic lateral sclerosis	Plasma	Prognosis/mortality risk	Small-RNA sequencing	I. Magen et al., 2021 [135]
miR-106a-5p	Relapsing-remitting multiple sclerosis	CSF	Association with early disease/inflammatory activity	RT-qPCR	A. Zanghì, et al., 2023 [136]
Multi-miRNA signature	Neurodegenerative and vascular-related disorders	Serum	Differential diagnostic classification	TaqMan RT-qPCR	C. Barbagallo et al., 2020 [137]
Exosomal miRNA profile	MCI/Alzheimer’s disease	Plasma-derived extracellular vesicles	Disease-stage discrimination	Small-RNA sequencing	X. Chen et al., 2025 [134]

MCI: Mild cognitive impairment.

## Data Availability

No new data were created or analyzed in this study. Data sharing is not applicable to this article.

## Abbreviations

The following abbreviations are used in this manuscript:

AD	Alzheimer’s disease
AGO	Argonaute
ALS	Amyotrophic lateral sclerosis
ASD	Autism spectrum disorder
BDNF	Brain-derived neurotrophic factor
bFGF	Basic fibroblast growth factor
BMP	Bone morphogenetic protein
CNS	Central nervous system
CREB	cAMP response element-binding protein
CREB1	cAMP response element-binding protein 1
CSF	Cerebrospinal fluid
DCX	Doublecortin
DGCR8	DiGeorge syndrome critical region 8
DHX36	DEAH-box helicase 36
DICER1	Dicer 1, ribonuclease III
DNA	Deoxyribonucleic acid
DROSHA	Drosha ribonuclease III
ERK	Extracellular signal-regulated kinase
EZH2	Enhancer of zeste homolog 2
FDA	U.S. Food and Drug Administration
FMRP	Fragile X messenger ribonucleoprotein
GABA	Gamma-aminobutyric acid
GTP	Guanosine triphosphate
HD	Huntington’s disease
HDAC4	Histone deacetylase 4
IDE	Insulin-degrading enzyme
IGF-1	Insulin-like growth factor 1
INSR	Insulin receptor
IRS-2	Insulin receptor substrate 2
LRH1	Liver receptor homolog 1
LSD1	Lysine-specific demethylase 1
MAPK	Mitogen-activated protein kinase
MCI	Mild cognitive impairment
miRNA	MicroRNA
miRISC	MicroRNA-induced silencing complex
mRNA	Messenger RNA
MS	Multiple sclerosis
MSK1	Mitogen- and stress-activated protein kinase 1
NGF	Nerve growth factor
NMDA	N-methyl-D-aspartate
NR2A	N-methyl-D-aspartate receptor subunit 2A
PAK	p21-activated kinase
PD	Parkinson’s disease
PI3K	Phosphoinositide 3-kinase
pre-miRNA	Precursor microRNA
pri-miRNA	Primary microRNA
REST	RE1-silencing transcription factor
RISC	RNA-induced silencing complex
RNA	Ribonucleic acid
ROCK1	Rho-associated coiled-coil-containing protein kinase 1
SCP1	Small C-terminal domain phosphatase 1
SIRT1	Sirtuin 1
TLX	Tailless nuclear receptor
TRBP	TAR RNA-binding protein
UTR	Untranslated region
XPO5	Exportin 5
